# Reversal of *MDR1*-associated resistance to topotecan by PAK-200S, a new dihydropyridine analogue, in human cancer cell lines

**DOI:** 10.1038/sj.bjc.6694384

**Published:** 1999-12

**Authors:** U Vanhoefer, M R Müller, R A Hilger, B Lindtner, U Klaassen, N Schleucher, Y M Rustum, S Seeber

**Affiliations:** Department of Internal Medicine (Cancer Research), West German Cancer Center, University of Essen Medical School, Essen 45122, Germany; Department of Pharmacology and Therapeutics, Grace Cancer Drug Center, Roswell Park Cancer Institute, Elm and Carlton Streets, Buffalo 14263, New York, USA

**Keywords:** topotecan, PAK-200S, multidrug resistance, breast cancer, ovarian cancer

## Abstract

Recent data suggest that expression of the membrane P_170_-glycoprotein (P-gp) may confer resistance to the topoisomerase- I-interactive agent topotecan. The present study describes the cellular effects of a new dihydropyridine analogue, PAK-200S, on P-gp-mediated resistance to topotecan in human breast and ovarian tumour cells. PAK-200S at a non-cytotoxic concentration of 2.0 μM completely reversed resistance to topotecan in P-gp-expressing MCF-7/adr (breast) and A2780/Dx5 (ovarian) tumour cells, respectively, with no effects on parental cells. Cellular pharmacokinetic studies by reversed-phase high-performance liquid chromatography analysis showed significantly lower cellular drug concentrations of the pharmacologically active closed-ring lactone of topotecan in multidrug-resistant cells than in parental cells. PAK-200S was effective in restoring the cellular lactone concentrations of topotecan in resistant MCF-7/adr cells to levels comparable to those obtained in parental cells. Furthermore, exposure of MCF-7/adr cells to topotecan in the presence of PAK-200S significantly increased the induction of protein-linked DNA breaks. PAK-200S did not alter nuclear topoisomerase I-mediated ex vivo pBR322 DNA plasmid unwinding activity and topoisomerase-I protein expression. These results suggest that reversal of P-gp-mediated resistance to topotecan by PAK-200S was related to the restoration of cellular drug concentrations of the active lactone form of topotecan rather than a direct effect on topoisomerase-I function. © 1999 Cancer Research Campaign


*British Journal of Cancer* (1999) **81**(8), 1304–1310
© 1999 Cancer Research Campaign
Article no. bjoc.1999.0845

### Reversal of *MDR1*-associated resistance to topotecan by PAK-200S, a new dihydropyridine analogue, in human cancer cell lines

**U Vanhoefer[1], MR Müller[1], RA Hilger[1], B Lindtner[1], U Klaassen[1], N Schleucher[1], YM Rustum[2], S Seeber[1] and A Harstrick[1]**

[1]Department of Internal Medicine (Cancer Research), West German Cancer Center, University of Essen Medical School, 45122 Essen, Germany; [2]Department of Pharmacology and Therapeutics, Grace Cancer Drug Center, Roswell Park Cancer Institute, Elm and Carlton Streets, Buffalo 14263, New York, USA

**Summary** Recent data suggest that expression of the membrane $P_{170}$-glycoprotein (P-gp) may confer resistance to the topoisomerase-I-interactive agent topotecan. The present study describes the cellular effects of a new dihydropyridine analogue, PAK-200S, on P-gp-mediated resistance to topotecan in human breast and ovarian tumour cells. PAK-200S at a non-cytotoxic concentration of 2.0 μM completely reversed resistance to topotecan in P-gp-expressing MCF-7/adr (breast) and A2780/Dx5 (ovarian) tumour cells, respectively, with no effects on parental cells. Cellular pharmacokinetic studies by reversed-phase high-performance liquid chromatography analysis showed significantly lower cellular drug concentrations of the pharmacologically active closed-ring lactone of topotecan in multidrug-resistant cells than in parental cells. PAK-200S was effective in restoring the cellular lactone concentrations of topotecan in resistant MCF-7/adr cells to levels comparable to those obtained in parental cells. Furthermore, exposure of MCF-7/adr cells to topotecan in the presence of PAK-200S significantly increased the induction of protein-linked DNA breaks. PAK-200S did not alter nuclear topoisomerase I-mediated ex vivo pBR322 DNA plasmid unwinding activity and topoisomerase-I protein expression. These results suggest that reversal of P-gp-mediated resistance to topotecan by PAK-200S was related to the restoration of cellular drug concentrations of the active lactone form of topotecan rather than a direct effect on topoisomerase-I function. © 1999 Cancer Research Campaign

**Keywords**: topotecan; PAK-200S; multidrug resistance; breast cancer; ovarian cancer

Topotecan (10-hydroxy-9-dimethyl-amino-methyl-camptothecin) is a new topoisomerase-I (TOP-I) targeting agent with significant clinical efficacy against a variety of solid tumours (Slichenmeyer et al, 1993; Rowinsky et al, 1997). Like other TOP-I interactive agents topotecan interacts with the breakage-rejoining reaction between DNA and TOP-I, generating intermediate forms of drug-stabilized covalent DNA–TOP-I complexes, referred to as cleavable complexes (Hsiang et al, 1988; Liu, 1989; Chen et al, 1994; Pommier et al, 1996). The cellular accumulation of such complexes may lead to cell death due to replication arrest and replication fork disassembly as well as chromosomal fragmentation (Covey et al, 1989; Ryan et al, 1991, 1994; Tsao et al, 1993). Like other camptothecin (CPT) analogues, topotecan establishes an equilibrium between the pharmacologically active closed lactone ring and the inactive open ring hydroxy acid form by reversible pH-dependent cellular hydrolysis (Grochow et al, 1992).

Acquired resistance to TOP-I interactive drugs has been related to down-regulation of TOP-I gene and protein expression (Eng et al, 1990; Kanzawa et al, 1990; Sugimoto et al, 1990; Kapoor et al, 1995; Sorensen et al, 1995) as well as alterations in TOP-I structure and function (e.g. point mutations, deletions and rearrangements of the TOP-I gene) (Andoh et al, 1987; Tan et al, 1989; Benedetti et al, 1993; Gromova et al, 1993). Current data suggest that positively charged CPT derivatives (e.g. topotecan) may be also transported by the *MDR1* gene product $P_{170}$-glycoprotein (P-gp), a $M_r$ 170 000 energy-dependent transmembrane glycoprotein efflux pump (Hendricks et al, 1992; Mattern et al, 1993; Maliepaard et al, 1996). Although altered cellular drug accumulation has been related to topotecan resistance, so far only few data are available on cellular pharmacokinetics of the pharmacologically active lactone form of topotecan and reversing agents of topotecan resistance in P-gp-expressing multidrug-resistant (MDR) cells (Hendricks et al, 1992).

The dihydropyridine analogue PAK-200S (2-[benzyl(phenyl)-amino]ethyl-1,4-dihydro-2,6-dimethyl-5-(5,5-dimethyl-2-oxo-1,3,2-dioxaphosphorinan-2-yl)-1-(2-morpholinoethyl)-4-(3-nitrophenyl)-3-pyridinecarboxylate) is an effective chemosensitizing modulator of membrane protein-associated drug resistance (P-gp and multidrug resistance protein [MRP]; U Vanhoefer et al, unpublished data) with a broad spectrum of activity in vitro and in vivo (Niwa et al, 1992). It has been shown that non-cytotoxic concentrations of PAK-200S inhibited [$^3$H]azidopine photolabelling of P-gp, associated with reversal of drug resistance in MDR KB-C2 and xenografted COK-36LN cells. An important feature in terms of clinical application of the dihydropyridine analogue PAK-200S is that this agent has the advantage over other calcium antagonists to exert effective P-gp modulatory properties with no significant calcium channel-blocking activity or other in vivo toxicity.

The present study describes the effects of the new dihydropyridine analogue PAK-200S on the cellular determinants of resistance

*Received 26 February 1999*
*Revised 11 June 1999*
*Accepted 16 June 1999*

*Correspondence to:* U Vanhoefer to topotecan in MDR human ovarian A2780/Dx5 and breast MCF-7/adr tumour cells that express the P-gp phenotype.

### MATERIALS AND METHODS

### Chemicals

Topotecan was obtained from SmithKline Beecham (King of Prussia, Philadelphia, PA, USA) and dissolved in dimethyl sulphoxide (DMSO) at a concentration of 10 mM (stock solution). PAK-200S was supplied by Nissan Chemical Ind. Co. (Chiba, Japan) and dissolved in DMSO (stock solution 10 mM). The highest concentration of DMSO used in all assays was found to be non-cytotoxic and without effect on the biochemical assays.

### Cell lines

The characteristics of the human ovarian cancer cell line A2780/wt (parental), the MDR subline A2780/Dx5 (P-gp-positive, MRP-negative), the human breast cancer cell line MCF-7/wt (parental) and the MDR subline MCF-7/adr (P-gp-positive, MRP-negative) have been reported previously (Fairchild et al, 1987; Minderman et al, 1996). Parental and resistant A2780 and MCF-7 cells were grown as monolayer in RPMI-1640 medium supplemented with 10% heat-inactivated fetal calf serum (FCS), and 2 mM L-glutamine. Cell cultures were kept in a humidified atmosphere of 5% carbon dioxide in air at 37°C. P-gp expression in A2780/Dx5 and MCF-7/adr cells was confirmed by flow cytometric analysis using monoclonal antibody (mAb) UIC2 (Müller et al, 1994). Representative histograms of single parameter green fluorescence plots showed a homogeneous population of MCF-7/adr cells staining for P-gp, while two subpopulations with different P-gp expression were found in the A2780/Dx5 cell line (data not shown) as already reported earlier (Minderman et al, 1996). Thus, MCF-7/wt and MCF-7/adr cells were used only for further biochemical analysis.

### In vitro cytotoxicity

Drug sensitivity was assessed by the sulphorhodamine B (SRB)-assay (Skehan et al, 1990). Exponentially growing human ovarian carcinoma A2780/wt and MDR A2780/Dx5 cells were seeded at a density of 1000 cells well$^{-1}$ in 96-well microtitre plates (Falcon, Becton-Dickinson Labware, Plymouth, UK) and allowed to attach overnight. Human breast cancer cells MCF-7/wt and MDR MCF-7/adr cells were seeded at a density of 800 cells well$^{-1}$. After 24 h cells were exposed for 24 h either to topotecan or to doxorubicin, respectively, washed twice with phosphate-buffered saline (PBS) and reincubated in drug-free medium. At four cell doubling times after beginning of drug treatment, cells were fixed with trichloric acetic acid, washed and stained with sulphorhodamine B as originally described. For modulation studies the cells were exposed for 24 h to the drug alone or in combination with a non-cytotoxic concentration (2.0 μM) of PAK-200S, washed twice with PBS and reincubated in drug-free medium with or without the modulator. The absorbance was measured at 570 nm using a 96-well plate reader (340 EL BIO Kinetics Reader, Bio-Tek Instruments Inc., Winooski, VT, USA). The drug concentrations which inhibited cell growth by 50% ($IC_{50}$) were determined from semilogarithmic dose–response plots.

### RP-HPLC-analysis of topotecan concentrations

Exponentially growing cells were trypsinized, washed twice with PBS and $2 \times 10^6$ cells per sample were resuspended in RPMI-1640 medium. Then, cells were exposed to drug-free medium or to medium containing 2.0 μM PAK-200S for 30 min at 37°C. For the measurements of cellular topotecan concentrations, 1.0 μM topotecan was added for the indicated time. For reversed-phase high-performance liquid chromatography (RP-HPLC) analysis cells were centrifuged at 11 000 ***g*** at 4°C, washed twice in ice-cold PBS buffer and resuspended in 500 μl of 0.1 M sodium citrate solution, and the extraction procedure was performed as described earlier (Beijnen et al, 1990). In brief, cells were lysed and deproteinized with 3 ml ice-cold methanol–acetonitrile (50:50, v/v), covered with nitrogen and frozen at –20°C for 12 h. After centrifugation at 1000 ***g*** for 15 min, supernatants were evaporated in a Speed Vac Plus Concentrator (Savant Instruments Inc., Farmingdale, NY, USA), resuspended in 200 μl of the mobile phase (methanol–phosphate buffer [60:40, v/v] adjusted to pH 5.9), covered with nitrogen and stored at –20°C for a further 2 h. The samples were centrifuged (21 900 ***g*** at 4°C for 5 min), and 50 μl of each sample were applied to HPLC. HPLC was performed with reversed-phase columns (4.6 × 250 mm) at 20°C packed with Nucleosil (C18) 5-μm particles (Machery and Nagel, Düren, Germany); sample injections were performed using a Waters 717 Plus Autosampler (Waters Inc., Eschborn, Germany). Absorbance of the column effluent was monitored using a fluorescence detector (Waters 470 Scanning Fluorescence Detector). Excitation was set to 370 nm and emission was adjusted to 525 nm. The peak areas were calculated using a Waters Millennium Chromatography Manager. Linearity was given to $r^2$ = 0.999 in the range of 5.0 μM–1.0 μM TPT, the limit of detection was 20 pM, the limit of quantitation was 50 pM (data not shown).

### TOP-I and TOP-II protein expression

Fifty micrograms of nuclear proteins were dissolved on a 7.5% sodium dodecyl sulphate (SDS) polyacrylamide gel and electroblotted to nitrocellulose (Bio-Rad, Hercules, CA, USA). For TOP-I immunoblotting, membranes were blocked with 5% (w/v) dried non-fat milk in PBS. TOP-I-specific binding of the human mAb against TOP-I (TopoGen Inc., Columbus, OH, USA) was visualized with the enhanced chemiluminescence (ECL) detection (Amersham Co., Arlington Heights, IL) using a protein A–horseradish peroxidase complex (Amersham Co.). TOP-II-specific binding of a rabbit polyclonal Ab (TopoGen Inc.) was visualized as mentioned above using a horseradish peroxidase complex.

### TOP-I unwinding activity of pBR322 plasmid DNA

Nuclear extracts were prepared according to the method of Danks et al (1988). In brief, $10^8$ cells were permeabilized in 5 mM potassium dihydrogen phosphate buffer pH 7.0 containing 2 mM magnesium chloride ($MgCl_2$), 4 mM dithiothreitol (DDT), 0.1 mM ethylenediaminetetraacetic acid (EDTA), and 1 mM phenylmethylsulphonyl fluoride (PMSF) (buffer A). Then, cells were centrifuged at 400 ***g*** for 5 min, and resuspended in buffer A containing 0.25 M sucrose, overlayed with buffer A containing 0.3 M sucrose and centrifuged at 2000 ***g*** for 20 min. The pellet was resuspended in 5 mM potassium dihydrogen phosphate buffer pH 7.0 containing 4 mM DTT, 1 mM EDTA and 1 mM PMSF. Nuclear

© 1999 Cancer Research Campaign

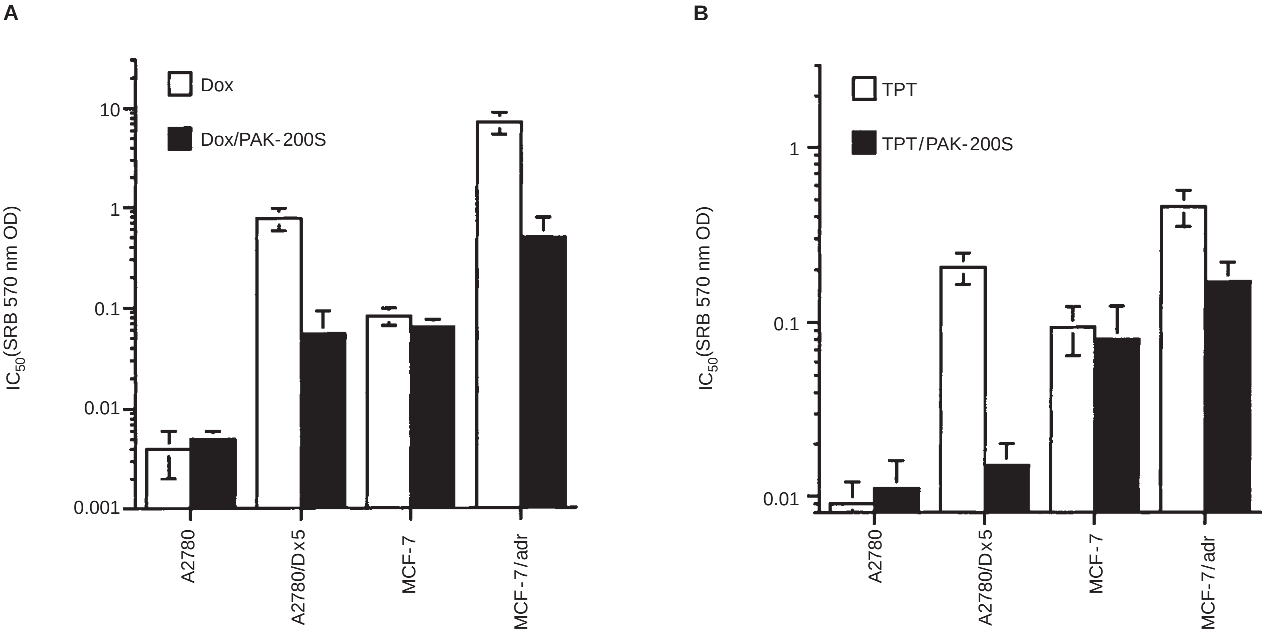

**Figure 1** Modulation of cellular resistance to doxorubicin (Dox) and topotecan (TPT) by a non-cytotoxic concentration of the dihydropyridine analogue PAK-200S in P-gp-expressing A2780/Dx5 and MCF-7/adr multidrug-resistant cells. Cytotoxicity was determined with the sulphorhodamine B (SRB) assay as described in Materials and Methods; all experiments were performed at least in triplicate proteins were eluted as 0.35 and 1.0 M salt extracts as originally described, washed and stored in aliquots at –80°C. To evaluate the effect of PAK-200S on the topotecan-mediated inhibition of ATP-independent unwinding of supercoiled pBR322 plasmid DNA ex vivo, a reaction mixture containing 0.3 μg $ml^{-1}$ pBR322 plasmid DNA and different dilutions of nuclear extract protein were incubated at exactly 37°C for 30 min with drug-free medium or with 50 μM topotecan ± 2.0 μM PAK-200S. The reaction products were separated on a 1.0% (w/v) agarose gel (4.6 V $cm^{-1}$) and stained with 0.5 μg $ml^{-1}$ ethidium bromide. Topotecan, DMSO and PAK-200S at the concentrations used had no effect on the pBR322 plasmid DNA (data not shown).

### Quantitation of protein-linked DNA breaks

The DNA of mid-logarithmic phase MCF-7/adr cells was labelled for 24 h with 0.1 μCi $ml^{-1}$ [$^{14}$C]dThy (DuPont, Boston, MA, USA; specific activity 62.8 mCi $mmol^{-1}$). Cells were harvested, washed and reincubated for 30 min at 37°C in [$^{14}$C]dThy-free medium containing 1.0–100 μM TPT with or without 2.0 μM PAK-200S. After drug exposure cells were washed in pre-warmed PBS (37°C) and the potassium chloride–SDS co-precipitation assay was immediately performed using 1 ml lysis buffer (1.25% SDS, 5 mM EDTA, 0.4 mg $ml^{-1}$ sheared salmon sperm DNA, adjusted to pH 8.0) per $10^6$ cells as described earlier (Trask et al, 1984; Vanhoefer et al, 1997). The results were calculated as percentage of precipitable [$^{14}$C]dThy-labelled DNA of drug-treated cells of total [$^{14}$C]dThy-DNA.

### Statistical analysis

The differences between the mean values were analysed for significance using the unpaired two-tailed Student's *t*-test for independent samples; $P$ values < 0.05 were considered to be statistically significant.

### RESULTS

### Cytotoxicity

The cytotoxicity of a 24-h drug exposure to doxorubicin (Figure 1A) and topotecan (Figure 1B) was investigated in parental and resistant MCF-7 and A2780 cells. MCF-7/adr and A2780/Dx5 cells showed 88-fold and 189-fold resistance to doxorubicin, whereas cross-resistance to topotecan was 5.6-fold and 23.6-fold, respectively. Cellular exposure to a non-cytotoxic concentration of PAK-200S (2 μM) partially reversed resistance to doxorubicin, but almost completely restored sensitivity to topotecan in both MDR cell lines (remaining resistance to topotecan in MCF-7/adr and A2780/Dx5 cells was 1.8-fold ($P > 0.05$) and 1.6-fold ($P > 0.05$), respectively) with no effect on parental MCF-7/wt and A2780/wt cells.

### Pharmacokinetics of cellular topotecan concentrations

The cellular topotecan concentrations in parental MCF-7/wt and P-gp-expressing MCF-7/adr cells were analysed by RP-HPLC and the results are shown in Figure 2. Cellular topotecan lactone concentrations in MCF-7/wt and MCF-7/adr cells were accumulated with steady-state levels at approximately 20 min. Resistant MCF-7/adr cells showed about 2.5-fold lower cellular lactone concentrations than parental MCF-7/wt cells with peak intracellular concentrations of 19.55 ± 3.19 pmol topotecan lactone $10^{-6}$ cells and 48.87 ± 3.92 pmol topotecan lactone $10^{-6}$ cells, respectively ($P < 0.01$). Exposure of MCF-7/adr cells to a non-cytotoxic concentration of 2.0 μM PAK-200S completely restored cellular lactone concentrations with 47.56 ± 2.98 pmol lactone $10^{-6}$ cells to the level observed for MCF-7/wt cells. PAK-200S had no effect on the drug concentrations in parental MCF-7/wt lacking P-gp expression (Figure 2). Similar results were observed in

© 1999 Cancer Research Campaign

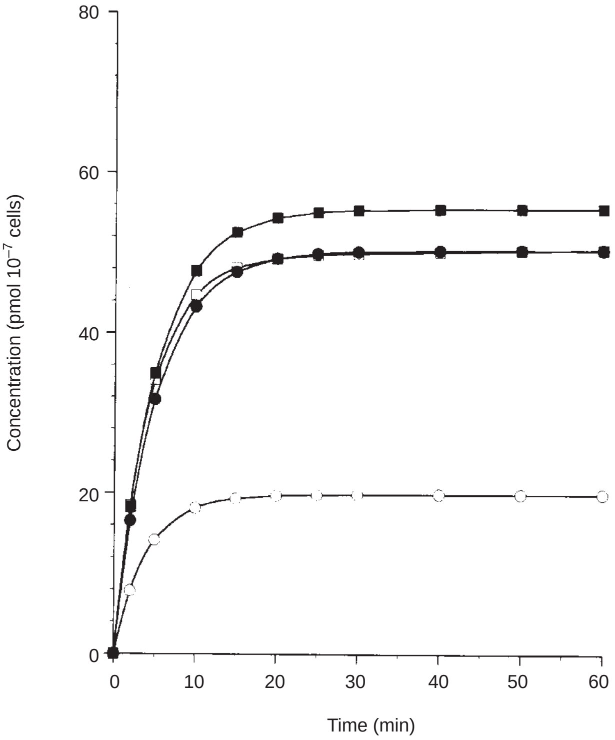

**Figure 2** Effect of the dihydropyridine analogue PAK-200S on the time-dependent cellular topotecan lactone concentrations in parental MCF-7/wt (rectangles) and resistant MCF-7/adr (circles) cells. Drug exposure and RP-HPLC analysis were performed as described in Materials and Methods. Cells were exposed to 1.0 μM TPT without (open symbols) or in the presence of 2.0 μM PAK-200S (closed symbols), respectively. ($P < 0.01$ for steady-state levels at approximately 20 min for MCF-7/adr cells with and without PAK-200S)

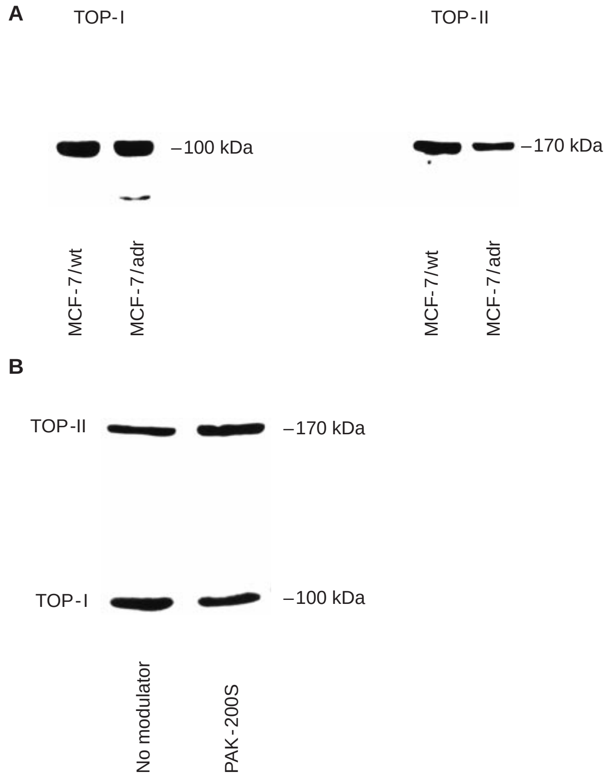

**Figure 3** Western blot analysis of TOP-I and -II protein expression in MCF-7/wt and MCF-7/adr cells (**A**). Fifty micrograms of nuclear proteins were dissolved on a 7.5% SDS-polyacrylamide gel and electroblotted to nitrocellulose. Binding of a specific human mAb against TOP-I was visualized with the enhanced chemiluminescence detection using a protein A–horseradish peroxidase complex, binding of a polyclonal rabbit Ab with TOP-II was visualized using a mouse anti-rabbit mAb, respectively. In addition, the effect of PAK-200S on TOP-I and -II protein expression in MCF-7/adr cells was determined (**B**). MCF-7/adr cells were exposed to 2.0 μM PAK-200S for 24 h prior to extraction of nuclear proteins parental A2780/wt and resistant A2780/Dx5 cells (data not shown).

### Immunoblot analysis of TOP-I and TOP-II expression

A representative TOP-I and TOP-II immunoblot of parental MCF-7/wt and resistant MCF-7/adr cells is shown in Figure 3A. Two bands were identified, the intact $M_r$ 100 000 TOP-I protein band and a $M_r$ 68 000 TOP-I proteolytic fragment (D'Arpa et al, 1988). No differences in TOP-I protein expression were found between topotecan-sensitive and -resistant MCF-7/wt cells. However, using a rabbit polyclonal antibody against TOP-II protein, MCF-7/adr cells revealed a minimally lower expression of TOP-II. Exposure of MCF-7/adr cells to 2 μM PAK-200S did not affect either TOP-I or TOP-II expression (Figure 3B).

### Induction of protein-linked DNA breaks

The maximum induction of protein-linked DNA breaks (PLDBs) by topotecan without or with PAK-200S was investigated in MCF-7/adr cells (Figure 4). After a 30-min exposure to increasing concentrations of topotecan, the induction of PLDBs increased in a dose-dependent manner (demonstrated as maximal induction of PLDBs), followed by a plateau phase. Exposure of MCF-7/adr cells to topotecan in the presence of a non-cytotoxic concentration of 2.0 μM PAK-200S significantly increased the maximum induction of PLDBs (PLDBs for 50 μM topotecan without or with 2.0 μM PAK-200S were 10.5 ± 2.1% vs 16.9 ± 4.4% ($P < 0.01$), respectively), demonstrating increased binding of topotecan to the nuclear DNA–TOP-I complex. No effect of PAK-200S on the maximum induction of PLDBs was found in parental MCF-7/wt cells (data not shown).

### TOP-I DNA unwinding activity

The effect of PAK-200S on the topotecan-mediated inhibition of the unwinding activity of TOP-I was investigated ex vivo using nuclear extracts of MCF-7/adr cells. The quantitation of ATP-independent TOP-I DNA unwinding activity was accomplished ex vivo by adding nuclear extracts to a reaction mixture containing supercoiled pBR322-DNA as substrate and the results are shown in Figure 5. Exposure of nuclear extracts to 50 μM of topotecan significantly inhibited TOP-I unwinding activity at 160 ng of nuclear proteins (Figure 5B). PAK-200S at 2.0 μM had no effect on the topotecan-mediated inhibition of TOP-I unwinding activity of pBR322 plasmid DNA (Figure 5C), results which indicate that PAK-200S does not affect the interactions between topotecan and TOP-I ex vivo.

*© 1999 Cancer Research Campaign*

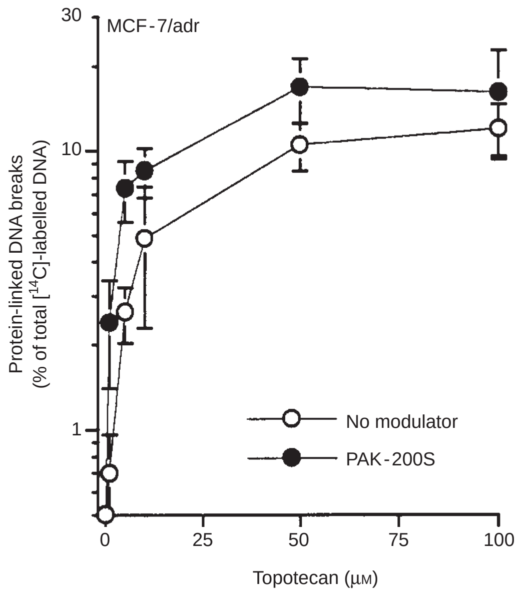

**Figure 4** Effect of PAK-200S on TPT-induced protein-linked DNA breaks (PLDBs) in resistant MCF-7/adr cells. Cells were exposed to different concentrations of TPT with or without 2.0 µM PAK-200S. Quantitation of PLDBs was performed by the KCl–SDS co-precipitation assay as described in Materials and Methods; results were calculated as percentage of precipitable [$^{14}$C]dThy-labelled DNA of total [$^{14}$C]dThy-DNA

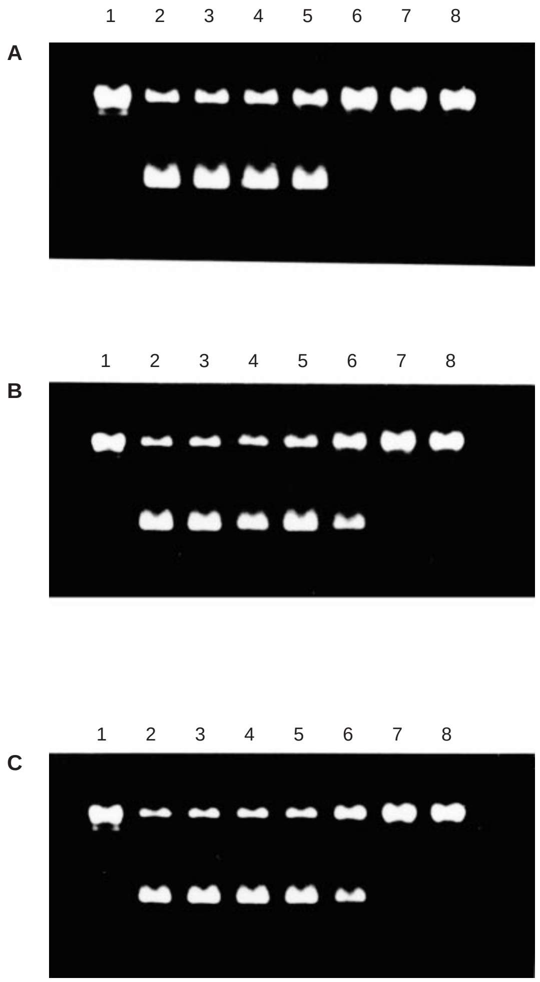

**Figure 5** Topotecan-mediated inhibition of TOP-I unwinding activity of pBR322-plasmid DNA ex vivo in the absence or presence of PAK-200S. Twenty to 640 ng of nuclear extract proteins of MCF-7/adr cells were incubated with a pBR322-plasmid DNA reaction mixture. pBR322 plasmid DNA with purified TOP-I enzyme [3 U] (lane 1); pBR322 plasmid DNA (lane 2); pBR322 plasmid DNA with 20 ng, 40 ng, 80 ng, 160 ng, 320 ng and 640 ng nuclear extract proteins (lanes 3–8). No drugs (**A**), 100 µM TPT (**B**), or 100 µM TPT and 2.0 µM PAK-200S (**C**), respectively

### DISCUSSION

The present study demonstrates that: (a) expression of the membrane transporter P-gp may result in decreased cellular drug concentrations of the pharmacologically active closed-ring lactone of topotecan, (b) dihydropyridine analogue PAK-200S, a new potent MDR-reversing agent, almost completely reverses resistance to topotecan in P-gp-expressing MCF-7/adr and A2780/Dx5 cells, with no effect on parental cells, and (c) reversal of topotecan resistance by PAK-200S is related to restoration of drug concentrations of the closed-ring lactone of topotecan rather than a direct effect on topoisomerases. Non-cytotoxic concentrations of PAK-200S (2.0 µM) completely restored cellular concentrations of the active lactone form of topotecan in MCF-7/adr cells (Figure 2), with no effect on parental cells lacking P-gp expression. Furthermore, restoration of cellular lactone concentrations by PAK-200S resulted in higher steady-state levels of PLDBs, the critical cytotoxic lesions of TOP-I interactive agents (Holm et al, 1989; Hsiang et al, 1989).

PAK-200S had no effect on the cellular TOP-I protein expression and enzyme activity (Figures 3B and 5) and no alterations of TOP-II expression were observed. These results are of importance because DNA topoisomerases I and II perform related functions, and TOP-II has been shown to compensate for TOP-I function (Uemura et al, 1984; Yang et al, 1987; Eng et al, 1990; Sugimoto et al, 1990; Friedman et al, 1994). The level of nuclear TOP-II is linked to cell proliferation, increasing during S phase and dropping to low levels during G1 phase (Markovits et al, 1987). TOP-II expression was slightly decreased in resistant MCF-7/adr cells (Figure 3A), however, no differences in cell doubling time were found between MCF-7/wt and MCF-7/adr cells (U Vanhoefer et al, unpublished data).

The reversible cellular hydrolysis of the pharmacologically active closed-ring lactone to the inactive open-ring hydroxy acid of topotecan has been shown to be pH-dependent (Grochow et al, 1992). PAK-200S at a concentration of 2.0 µM showed no effect on the pH of whole cell lysates of MCF-7/adr cells. In addition, no differences were found between MCF-7/wt and resistant MCF-7/adr cells in terms of cellular pH (data not shown). Based on these data, reversal of topotecan resistance by PAK-200S is likely related to inhibition of P-gp function in MCF-7/adr cells.

Previous studies on drug-selected P-gp-expressing MDR cells (Hendricks et al, 1992; Mattern et al, 1993; Maliepaard et al, 1996) and *MDR1* cDNA-transfected NIH 3T3 cells (Hoki et al, 1997) showed that expression of P-gp may confer resistance to positively charged TOP-I interactive agents (e.g. topotecan). Furthermore, inhibition of P-gp function by quinidine resulted in an increased accumulation of topotecan in MCF-7/adria$^{r}$ and KG1a cells (Hendricks et al, 1992). Of importance is that irinotecan (CPT-11; [7-ethyl-10-[4-(1-piperidino)-1-piperidino]-carbonyloxy-CPT]) and its active metabolite SN-38 (10-hydroxy-7-ethyl-campto-

 © 1999 Cancer Research Campaign thecin) appear not to be substrates for P-gp-mediated drug resistance in vitro and in vivo (Jansen et al, 1998). In addition, P-gp- and MRP-independent mechanisms of membrane protein-associated resistance to topotecan have been observed (Yang et al, 1995; Ma et al, 1998). Yang and co-workers (1995) reported in a mitoxantrone-resistant cell line an energy-dependent efflux of topotecan associated with a high level of drug resistance and reduced steady-state levels of topotecan-mediated TOP-I enzyme/DNA complexes. In the present study, PAK-200S restored cellular concentrations of topotecan in MCF-7/adr cells, which was associated with an increased induction of protein-linked DNA breaks. However, additional TOP-I specific effects of PAK-200S as mechanisms of chemosensitization to topotecan in MCF-7/adr cells cannot be excluded and need further investigation.

The dihydropyridine analogue PAK-200S almost completely restored drug response to topotecan in human MCF-7/adr breast and A2780/Dx5 ovarian MDR cells, whereas resistance to doxorubicin was only partially reversed, results that are likely related to the fact that P-gp-mediated resistance to topotecan appears to be evident to a considerably lesser extent than that observed for other P-gp substrates (e.g. taxanes, anthracyclines). PAK-104P, a structurally related pyridine analogue, reversed in vitro and in vivo MRP-mediated multidrug resistance associated with an inhibition of the ATP-dependent [$^3$H]leukotriene C4 transport in membrane vesicles of MRP-expressing CA120 cells (Vanhoefer et al, 1996; Chuman et al, 1997). In addition, data from our laboratory showed that PAK-200S at non-cytotoxic concentrations significantly reversed MRP-mediated 250-fold resistance to doxorubicin in MRP-expressing human fibrosarcoma HT1080/Dr4 cells. Since recent data suggest that MRP may also confer resistance to topotecan in vitro (Jonsson et al, 1997; Jansen et al, 1998) and in vivo (U Vanhoefer et al, unpublished data), PAK-200S may have clinical advantages over other MDR-reversing agents (e.g. PSC-833), lacking significant inhibition of MRP-function (Twentyman et al, 1994; Vanhoefer et al, 1996).

Based on the data reported herein, the dihydropyridine analogue PAK-200S is a potent reversing agent of membrane protein-associated resistance to topotecan and therefore merits further preclinical and clinical investigation.

### ACKNOWLEDGEMENTS

U Vanhoefer, MR Müller and A Harstrick are supported by the Deutsche Forschungsgemeinschaft grant RA 119/17-2. We thank Dr Hans Minderman (Grace Cancer Drug Center, Roswell Park Cancer Institute, Buffalo, New York) for the critical reading of the manuscript.

### REFERENCES

Andoh T, Ishii K, Suzuki Y, Ikegami Y, Kusunoki Y, Takemoto Y and Okada K (1987) Characterization of a mammalian mutant with a camptothecin-resistant DNA topoisomerase I. *Proc Natl Acad Sci USA* **84**: 5565–5569

Beijnen JH, Smith BR, Keijer WJ, van Gijn R, ten Bokkel Huinink WW, Vlasveld LT, Rodenhuis S and Underberg WJ (1990) High-performance liquid chromatographic analysis of the new antitumor drug SK&F 104864-A (NSC 609699) in plasma. *J Pharm Biomed Anal* **8**: 789–795

Benedetti P, Fiorani P, Capuani L and Wang JC (1993) Camptothecin resistance from a single mutation changing glycine 363 of human DNA topoisomerase I to cysteine. *Cancer Res* **53**: 4343–4348

Chen AY and Liu LF (1994) DNA topoisomerases: essential enzymes and lethal targets. *Annu Rev Pharmacol Toxicol* **34**: 191–218

Chuman Y, Chen ZS, Sumizawa T, Seto K, Furukawa T, Haraguchi M, Tani A, Shudo N, Yamada K, Akiyama S and Aikou T (1997) Reversal of MRP-associated drug resistance by the pyridine analogue, PAK-104P. *Proc Am Assoc Cancer Res* **38**: 594 (abstract)

Covey JM, Jaxel C, Kohn KW and Pommier Y (1989) Protein-linked DNA strand breaks induced in mammalian cells by camptothecin, an inhibitor of topoisomerase I. *Cancer Res* **49**: 5016–5022

Danks MK, Schmidt CA, Cirtain MC, Suttle P and Beck WT (1988) Altered catalytic activity of and DNA cleavable by DNA topoisomerase II from human leukemic cells selected for resistance to VM-26. *Biochemistry* **27**: 8861–8869

D'Arpa P, Machlin PS, Ratrie H III, Rothfield NF, Cleveland DW and Earnshaw WC (1988) cDNA cloning of human DNA topoisomerase I: catalytic activity of a 67.7-kDa carboxyl-terminal fragment. *Proc Natl Acad Sci USA* **85**: 2543–2547

Eng WK, McCabe FL, Tan KB, Mattern MR, Hofmann GA, Woessner RD, Hertzberg RP and Johnson RK (1990) Development of a stable camptothecin-resistant subline of P388 leukemia with reduced topoisomerase I content. *Mol Pharmacol* **38**: 471–480

Fairchild CR, Ivy SP, Kao-Shan CS, Whang-Peng J, Rosen N, Israel MA, Melera PW, Cowan KH and Goldsmith ME (1987) Isolation of amplified and overexpressed DNA sequences from adriamycin-resistant human breast cancer cells. *Cancer Res* **47**: 5141–5148

Friedman HS, Dolan ME, Kaufmann SH, Colvin OM, Griffith OW, Moschel RC, Schold SC, Bigner DD and Ali-Osman F (1994) Elevated DNA polymerase α, DNA polymerase β, and DNA topoisomerase II in a melphalan-resistant rhabdomyosarcoma xenograft that is cross-resistant to nitrosoureas and topotecan. *Cancer Res* **54**: 3487–3493

Grochow LB, Rowinsky EK, Johnson R, Ludeman S, Kaufmann SH, McCabe FL, Smith BR, Hurowitz L, DeLisa A, Donehower RC and Noe DA (1992) Pharmacokinetics and pharmacodynamics of topotecan in patients with advanced cancer. *Drug Metab Dispos Fate Biol Chem* **20**: 706–713

Gromova I, Kjeldsen E, Svejstrup JQ, Alsner Q, Christiansen K and Westergaard O (1993) Characterization of an altered DNA catalysis of a camptothecin-resistant eukaryotic topoisomerase I. *Nucleic Acids Res* **21**: 593–600

Hendricks CB, Rowinsky EK, Grochow LB, Donehower RC and Kaufmann SH (1992) Effect of P-glycoprotein expression on the accumulation and cytotoxicity of topotecan (SK&F 104864), a new camptothecin analogue. *Cancer Res* **52**: 2268–2278

Hoki Y, Fujimori A and Pommier Y (1997) Differential cytotoxicity of clinically important camptothecin derivatives in P-glycoprotein-overexpressing cell lines. *Cancer Chemother Pharmacol* **40**: 433–438

Holm C, Covey JM, Kerrigan D and Pommier Y (1989) Differential requirement of DNA replication for the cytotoxicity of DNA topoisomerase I and II inhibitors in Chinese hamster DC3F cells. *Cancer Res* **49**: 6365–6368

Hsiang Y-H and Liu LF (1988) Identification of mammalian DNA topoisomerase I as an intracellular target of the anticancer drug camptothecin. *Cancer Res* **48**: 1722–1726

Hsiang Y-H, Lihou MG and Liu LF (1989) Arrest of replication forks by drug-stabilized topoisomerase I-DNA cleavable complexes as a mechanism of cell killing by camptothecin. *Cancer Res* **49**: 5077–5082

Jansen WJM, Hulscher TM, van Ark-Otte J, Giaccone G, Pinedo HM and Boven E (1998) CPT-11 sensitivity in relation to the expression of P170-glycoprotein and multidrug resistance-associated protein. *Br J Cancer* **77**: 359–365

Jonsson E, Fridborg H, Csóka K, Dhar S, Sundström C, Nygren P and Larsson R (1997) Cytotoxic activity of topotecan in human tumour cell lines and primary cultures of human tumour cells from patients. *Br J Cancer* **76**: 211–219

Kanzawa F, Sugimoto Y, Minato K, Kasahara K, Bungo M, Nakagawa K, Fujiwara Y, Liu LF and Saijo N (1990) Establishment of a camptothecin analogue (CPT-11)-resistant cell line of human non-small cell lung cancer: characterization and mechanism of resistance. *Cancer Res* **50**: 5919–5924

Kapoor R, Slade DL, Fujimori A, Pommier Y and Harker WG (1995) Altered topoisomerase I expression in two subclones of human CEM leukemia selected for resistance to camptothecin. *Oncol Res* **7**: 83–95

Liu LF (1989) DNA topoisomerase poisons as antitumor drugs. *Annu Rev Biochem* **58**: 351–375

Ma J, Maliepaard M, Nooter K, Loos WJ, Kolker HJ, Verweij J, Stoter G and Schellens JHM (1998) Reduced cellular accumulation of topotecan: a novel mechanism of resistance in a human ovarian cancer cell line. *Br J Cancer* **77**: 1645–1652

Maliepaard M, Nooter K, Ma J, Loos WJ, Kolker HJ, Verweij J, Stoter G and Schellens JHM (1996) Relationship between P-glycoprotein, multidrug resistance-associated protein, and cellular accumulation of topoisomerase I inhibitors. *Proc Am Assoc Cancer Res* **37**: 312 (abstract)

Markovits J, Pommier Y, Kerrigan D, Covey JM, Tilchen EJ and Kohn KW (1987) Topoisomerase II-mediated DNA breaks and cytotoxicity in relation to cell proliferation and the cell cycle in NIH 3T3 fibroblasts and L1210 leukemia cells. *Cancer Res* **47**: 2050–2055

Mattern MR, Hofmann GA, Polsky RM, Funk LR, McCabe FL and Johnson FL (1993) In vitro and in vivo effects of clinically important camptothecin analogues on multidrug-resistant cells. *Oncol Res* **5**: 467–474

Minderman H, Vanhoefer U, Toth K, Minderman MD and Rustum YM (1996) A unique human ovarian carcinoma cell line expressing CD34 in association with selection for multidrug resistance. *Cancer* **78**: 2428–2436

Müller MR, Lennartz K, Nowrousian MR, Dux R, Tsuruo T, Rajewsky MF and Seeber S (1994) Improved flow-cytometric detection of low P-glycoprotein expression in leukaemic blasts by histogram subtraction analysis. *Cytometry* **15**: 64–72

Niwa K, Yamada K, Furukawa T, Shudo N, Seto K, Matsumoto T, Takao S, Akiyama S and Shimazu H (1992) Effect of a dihydropyridine analogue, 2-[benzyl(phenyl)-amino]ethyl 1,4-dihydro-2,6-dimethyl-5-(5,5-dimethyl-2-oxo-1,3,2-dioxaphosphorinan-2-yl)-1-(2-morpholinoethyl)-4-(3-nitrophenyl)-3-pyridinecarboxylate on reversing in vivo resistance of tumor cells to adriamycin. *Cancer Res* **52**: 3655–3660

Pommier Y (1996) Eukaryotic DNA topoisomerase I: genome gatekeeper and its intruders, camptothecins. *Semin Oncol* **23** (suppl 3): 3–10

Rowinsky EK and Verweij J (1997) Review of phase I clinical studies with topotecan. *Semin Oncol* **24** (suppl 20): 3–10

Ryan AJ, Squires S, Strutt HL and Johnson RT (1991) Camptothecin cytotoxicity in mammalian cells is associated with the induction of persistent double strand breaks in replicating DNA. *Nucleic Acids Res* **19**: 3295–3300

Ryan AJ, Squires S, Strutt HL, Evans A and Johnson RT (1994) Different fates of camptothecin-induced replication fork-associated double-strand DNA breaks in mammalian cells. *Carcinogenesis* **15**: 823–828

Skehan P, Storeng R, Scudiero D, Monks A, McMahon J, Vistica D, Warren JT, Bokesch H, Kenney S and Boyd MR (1990) New colorimetric cytotoxicity assay for anticancer drug screening. *J Natl Cancer Inst* **82**: 1107–1112

Slichenmeyer WJ, Rowinsky EK, Donehower RC and Kaufmann SH (1993) The current status of camptothecin analogues as antitumor agents. *J Natl Cancer Inst* **85**: 271–291

Sorenson M, Sehested M and Jensen PB (1995) Characterisation of a human small-cell lung cancer cell line resistant to the DNA topoisomerase I-directed drug topotecan. *Br J Cancer* **72**: 399–404

Sugimoto Y, Tsukahara S, Oh-Hara T, Isoe T and Tsuruo T (1990*a*) Decreased expression of DNA topoisomerase I in camptothecin-resistant tumor cell lines as determined by a monoclonal antibody. *Cancer Res* **50**: 6925–6930.

Sugimoto Y, Tsukahara S, Oh-Hara T, Liu LF and Tsuruo T (1990*b*) Elevated expression of DNA topoisomerase II in camptothecin-resistant human tumor cell lines. *Cancer Res* **50**: 7962–7965

Tan KB, Mattern MR, Eng WK, McCabe FL and Johnson RK (1989) Nonproductive rearrangement of DNA topoisomerase I and II genes: correlation with resistance to topoisomerase inhibitors. *J Natl Cancer Inst* **81**: 1732–1735

Trask DK, DiDonato JA and Muller MT (1984) Rapid detection and isolation of covalent DNA/protein complexes: application to topoisomerase I and II. *EMBO* **3**: 671–676.

Tsao YP, Russo A, Nyamuswa G, Silber R and Liu LF (1993) Interaction between replication forks and topoisomerase I-DNA cleavable complexes: studies in a cell-free SV40 DNA replication system. *Cancer Res* **53**: 5908–5914

Twentyman PR, Wright KA and Bleehen NM (1994) Modification by SDZ PSC-833 of sensitivity to etoposide, cisplatin and taxol in multidrug resistant (MDR) cells with P-glycoprotein (Pgp)-mediated and MRP-associated phenotypes. *Ann Oncol* **5** (Suppl 5): 153 (abstract)

Uemura T and Yanagida M (1984) Isolation of type I and II DNA topoisomerase mutants from fission yeast: single and double mutants show different phenotypes in cell growth and chromatin organization. *EMBO* **3**: 1734–1744

Vanhoefer U, Cao S, Minderman H, Toth K, Scheper RJ, Slovak ML and Rustum YM (1996) PAK-104P, a pyridine analogue, reverses paclitaxel and doxorubicin resistance in cell lines and nude mice bearing xenografts that overexpress the multidrug resistance protein. *Clin Cancer Res* **2**: 369–377

Vanhoefer U, Voigt W, Hilger RA, Yin MB, Harstrick A, Seeber S and Rustum YM (1997) Cellular determinants of resistance to indolocarbazole analogue 6-N-formylamino-12,13-dihydro-1,11-dihydroxy-13 (β-D-glucopyranosyl)-5H-indolo[2,3-alpha]pyrrolo[3,4-c]carbazole-5,7(6H)-dione [NB-506], a novel potent topoisomerase I inhibitor, in multidrug-resistant human tumor cells. *Oncol Res* **9**: 485–494

Yang C-HJ, Horton JK, Cowan KH and Schneider E (1995) Cross-resistance to camptothecin analogues in a mitoxantrone-resistant human breast carcinoma cell line is not due to DNA topoisomerase I alterations. *Cancer Res* **55**: 4004–4009

Yang L, Wold MS, Li JJ, Kelly TJ and Liu LF (1987) Roles of DNA topoisomerases in simian virus 40 DNA replication in vitro. *Proc Natl Acad Sci USA* **84**: 950–954

 *© 1999 Cancer Research Campaign*

## References

[PDF_00537] Andoh T., Ishii K., Suzuki Y., Ikegami Y., Kusunoki Y., Takemoto Y., Okada K. (1987). Characterization of a mammalian mutant with a camptothecin-resistant DNA topoisomerase I.. Proc Natl Acad Sci U S A.

[PDF_00540] Beijnen J. H., Smith B. R., Keijer W. J., van Gijn R., ten Bokkel Huinink W. W., Vlasveld L. T., Rodenhuis S., Underberg W. J. (1990). High-performance liquid chromatographic analysis of the new antitumour drug SK&F 104864-A (NSC 609699) in plasma.. J Pharm Biomed Anal.

[PDF_00544] Benedetti P., Fiorani P., Capuani L., Wang J. C. (1993). Camptothecin resistance from a single mutation changing glycine 363 of human DNA topoisomerase I to cysteine.. Cancer Res.

[PDF_00547] Chen A. Y., Liu L. F. (1994). DNA topoisomerases: essential enzymes and lethal targets.. Annu Rev Pharmacol Toxicol.

[PDF_00553] Covey J. M., Jaxel C., Kohn K. W., Pommier Y. (1989). Protein-linked DNA strand breaks induced in mammalian cells by camptothecin, an inhibitor of topoisomerase I.. Cancer Res.

[PDF_00559] D'Arpa P., Machlin P. S., Ratrie H., Rothfield N. F., Cleveland D. W., Earnshaw W. C. (1988). cDNA cloning of human DNA topoisomerase I: catalytic activity of a 67.7-kDa carboxyl-terminal fragment.. Proc Natl Acad Sci U S A.

[PDF_00556] Danks M. K., Schmidt C. A., Cirtain M. C., Suttle D. P., Beck W. T. (1988). Altered catalytic activity of and DNA cleavage by DNA topoisomerase II from human leukemic cells selected for resistance to VM-26.. Biochemistry.

[PDF_00562] Eng W. K., McCabe F. L., Tan K. B., Mattern M. R., Hofmann G. A., Woessner R. D., Hertzberg R. P., Johnson R. K. (1990). Development of a stable camptothecin-resistant subline of P388 leukemia with reduced topoisomerase I content.. Mol Pharmacol.

[PDF_00566] Fairchild C. R., Ivy S. P., Kao-Shan C. S., Whang-Peng J., Rosen N., Israel M. A., Melera P. W., Cowan K. H., Goldsmith M. E. (1987). Isolation of amplified and overexpressed DNA sequences from adriamycin-resistant human breast cancer cells.. Cancer Res.

[PDF_00570] Friedman H. S., Dolan M. E., Kaufmann S. H., Colvin O. M., Griffith O. W., Moschel R. C., Schold S. C., Bigner D. D., Ali-Osman F. (1994). Elevated DNA polymerase alpha, DNA polymerase beta, and DNA topoisomerase II in a melphalan-resistant rhabdomyosarcoma xenograft that is cross-resistant to nitrosoureas and topotecan.. Cancer Res.

[PDF_00575] Grochow L. B., Rowinsky E. K., Johnson R., Ludeman S., Kaufmann S. H., McCabe F. L., Smith B. R., Hurowitz L., DeLisa A., Donehower R. C. (1992). Pharmacokinetics and pharmacodynamics of topotecan in patients with advanced cancer.. Drug Metab Dispos.

[PDF_00579] Gromova I. I., Kjeldsen E., Svejstrup J. Q., Alsner J., Christiansen K., Westergaard O. (1993). Characterization of an altered DNA catalysis of a camptothecin-resistant eukaryotic topoisomerase I.. Nucleic Acids Res.

[PDF_00582] Hendricks C. B., Rowinsky E. K., Grochow L. B., Donehower R. C., Kaufmann S. H. (1992). Effect of P-glycoprotein expression on the accumulation and cytotoxicity of topotecan (SK&F 104864), a new camptothecin analogue.. Cancer Res.

[PDF_00586] Hoki Y., Fujimori A., Pommier Y. (1997). Differential cytotoxicity of clinically important camptothecin derivatives in P-glycoprotein-overexpressing cell lines.. Cancer Chemother Pharmacol.

[PDF_00589] Holm C., Covey J. M., Kerrigan D., Pommier Y. (1989). Differential requirement of DNA replication for the cytotoxicity of DNA topoisomerase I and II inhibitors in Chinese hamster DC3F cells.. Cancer Res.

[PDF_00595] Hsiang Y. H., Lihou M. G., Liu L. F. (1989). Arrest of replication forks by drug-stabilized topoisomerase I-DNA cleavable complexes as a mechanism of cell killing by camptothecin.. Cancer Res.

[PDF_00592] Hsiang Y. H., Liu L. F. (1988). Identification of mammalian DNA topoisomerase I as an intracellular target of the anticancer drug camptothecin.. Cancer Res.

[PDF_00598] Jansen W. J., Hulscher T. M., van Ark-Otte J., Giaccone G., Pinedo H. M., Boven E. (1998). CPT-11 sensitivity in relation to the expression of P170-glycoprotein and multidrug resistance-associated protein.. Br J Cancer.

[PDF_00601] Jonsson E., Fridborg H., Csóka K., Dhar S., Sundström C., Nygren P., Larsson R. (1997). Cytotoxic activity of topotecan in human tumour cell lines and primary cultures of human tumour cells from patients.. Br J Cancer.

[PDF_00604] Kanzawa F., Sugimoto Y., Minato K., Kasahara K., Bungo M., Nakagawa K., Fujiwara Y., Liu L. F., Saijo N. (1990). Establishment of a camptothecin analogue (CPT-11)-resistant cell line of human non-small cell lung cancer: characterization and mechanism of resistance.. Cancer Res.

[PDF_00608] Kapoor R., Slade D. L., Fujimori A., Pommier Y., Harker W. G. (1995). Altered topoisomerase I expression in two subclones of human CEM leukemia selected for resistance to camptothecin.. Oncol Res.

[PDF_00611] Liu L. F. (1989). DNA topoisomerase poisons as antitumor drugs.. Annu Rev Biochem.

[PDF_00613] Ma J., Maliepaard M., Nooter K., Loos W. J., Kolker H. J., Verweij J., Stoter G., Schellens J. H. (1998). Reduced cellular accumulation of topotecan: a novel mechanism of resistance in a human ovarian cancer cell line.. Br J Cancer.

[PDF_00629] Mattern M. R., Hofmann G. A., Polsky R. M., Funk L. R., McCabe F. L., Johnson R. K. (1993). In vitro and in vivo effects of clinically important camptothecin analogues on multidrug-resistant cells.. Oncol Res.

[PDF_00632] Minderman H., Vanhoefer U., Toth K., Minderman M. D., Rustum Y. M. (1996). A unique human ovarian carcinoma cell line expressing CD34 in association with selection for multidrug resistance.. Cancer.

[PDF_00635] Müller M. R., Lennartz K., Nowrousian M. R., Dux R., Tsuruo T., Rajewsky M. F., Seeber S. (1994). Improved flow-cytometric detection of low P-glycoprotein expression in leukaemic blasts by histogram subtraction analysis.. Cytometry.

[PDF_00639] Niwa K., Yamada K., Furukawa T., Shudo N., Seto K., Matsumoto T., Takao S., Akiyama S., Shimazu H. (1992). Effect of a dihydropyridine analogue, 2-[benzyl(phenyl)amino]ethyl 1,4-dihydro-2,6-dimethyl-5-(5,5-dimethyl-2-oxo- 1,3,2-dioxaphosphorinan-2-yl)-1-(2-morpholinoethyl)-4-(3-nitrophenyl)-3 -pyridinecarboxylate on reversing in vivo resistance of tumor cells to adriamycin.. Cancer Res.

[PDF_00645] Pommier Y. (1996). Eukaryotic DNA topoisomerase I: genome gatekeeper and its intruders, camptothecins.. Semin Oncol.

[PDF_00652] Ryan A. J., Squires S., Strutt H. L., Evans A., Johnson R. T. (1994). Different fates of camptothecin-induced replication fork-associated double-strand DNA breaks in mammalian cells.. Carcinogenesis.

[PDF_00649] Ryan A. J., Squires S., Strutt H. L., Johnson R. T. (1991). Camptothecin cytotoxicity in mammalian cells is associated with the induction of persistent double strand breaks in replicating DNA.. Nucleic Acids Res.

[PDF_00647] Sandler D. P., Ross J. A. (1997). Epidemiology of acute leukemia in children and adults.. Semin Oncol.

[PDF_00655] Skehan P., Storeng R., Scudiero D., Monks A., McMahon J., Vistica D., Warren J. T., Bokesch H., Kenney S., Boyd M. R. (1990). New colorimetric cytotoxicity assay for anticancer-drug screening.. J Natl Cancer Inst.

[PDF_00658] Slichenmyer W. J., Rowinsky E. K., Donehower R. C., Kaufmann S. H. (1993). The current status of camptothecin analogues as antitumor agents.. J Natl Cancer Inst.

[PDF_00661] Sorensen M., Sehested M., Jensen P. B. (1995). Characterisation of a human small-cell lung cancer cell line resistant to the DNA topoisomerase I-directed drug topotecan.. Br J Cancer.

[PDF_00664] Sugimoto Y., Tsukahara S., Oh-hara T., Isoe T., Tsuruo T. (1990). Decreased expression of DNA topoisomerase I in camptothecin-resistant tumor cell lines as determined by a monoclonal antibody.. Cancer Res.

[PDF_00667] Sugimoto Y., Tsukahara S., Oh-hara T., Liu L. F., Tsuruo T. (1990). Elevated expression of DNA topoisomerase II in camptothecin-resistant human tumor cell lines.. Cancer Res.

[PDF_00670] Tan K. B., Mattern M. R., Eng W. K., McCabe F. L., Johnson R. K. (1989). Nonproductive rearrangement of DNA topoisomerase I and II genes: correlation with resistance to topoisomerase inhibitors.. J Natl Cancer Inst.

[PDF_00673] Trask D. K., DiDonato J. A., Muller M. T. (1984). Rapid detection and isolation of covalent DNA/protein complexes: application to topoisomerase I and II.. EMBO J.

[PDF_00676] Tsao Y. P., Russo A., Nyamuswa G., Silber R., Liu L. F. (1993). Interaction between replication forks and topoisomerase I-DNA cleavable complexes: studies in a cell-free SV40 DNA replication system.. Cancer Res.

[PDF_00683] Uemura T., Yanagida M. (1984). Isolation of type I and II DNA topoisomerase mutants from fission yeast: single and double mutants show different phenotypes in cell growth and chromatin organization.. EMBO J.

[PDF_00686] Vanhoefer U., Cao S., Minderman H., Tóth K., Scheper R. J., Slovak M. L., Rustum Y. M. (1996). PAK-104P, a pyridine analogue, reverses paclitaxel and doxorubicin resistance in cell lines and nude mice bearing xenografts that overexpress the multidrug resistance protein.. Clin Cancer Res.

[PDF_00690] Vanhoefer U., Voigt W., Hilger R. A., Yin M. B., Harstrick A., Seeber S., Rustum Y. M. (1997). Cellular determinants of resistance to indolocarbazole analogue 6-N-formylamino-12,13-dihydro-1,11-dihydroxy-13(beta-D-glucopyranosyl)- 5H-indolo[2,3-alpha]pyrrolo[3,4-c]carbazole-5,7(6H)-dione (NB-506), a novel potent topoisomerase I inhibitor, in multidrug-resistant human tumor cells.. Oncol Res.

[PDF_00696] Yang C. J., Horton J. K., Cowan K. H., Schneider E. (1995). Cross-resistance to camptothecin analogues in a mitoxantrone-resistant human breast carcinoma cell line is not due to DNA topoisomerase I alterations.. Cancer Res.

[PDF_00700] Yang L., Wold M. S., Li J. J., Kelly T. J., Liu L. F. (1987). Roles of DNA topoisomerases in simian virus 40 DNA replication in vitro.. Proc Natl Acad Sci U S A.

